# Supplementary material for: CD115− monocytic myeloid-derived suppressor cells are precursors of OLFM4high polymorphonuclear myeloid-derived suppressor cells
Source: Commun Biol. 2023 Mar 15;6:272. doi: 10.1038/s42003-023-04650-3 (PMC10017706; doi:10.1038/s42003-023-04650-3)
Supplement: Supplementary file 2 — Supplementary information [file 42003_2023_4650_MOESM2_ESM.pdf]

Supplementary Information for

CD115$^-$ monocytic myeloid-derived suppressor cells are precursors of OLFM4^{high} polymorphonuclear myeloid-derived suppressor cells

Yunyun Zou,[1,2] Nobuhiko Kamada,[3] Seung-Yong Seong,[1,2]* Sang-Uk Seo[4]*

[1] Department of Biomedical Sciences, Seoul National University College of Medicine, Seoul, Republic of Korea.

[2] Wide River Institute of Immunology, Seoul National University College of Medicine, Hongcheon, Republic of Korea.

[3] Division of Gastroenterology and Hepatology, Department of Internal Medicine, University of Michigan, Ann Arbor, Michigan, USA.

[4] Department of Microbiology, College of Medicine, The Catholic University of Korea, Seoul, Republic of Korea

Correspondence:

Sang-Uk Seo

Address: 222 Banpo-daero, Seocho-gu, Seoul 06591, Republic of Korea

Work: +82-2-3147-8367

Mobile: +82-10-4722-9930

Email: suseo@catholic.ac.kr

Seung-Yong Seong

Address: 103 Daehak-ro, Jongno-gu, Seoul, 03080, Republic of Korea

Work: +82-2-740-8301

Mobile: +82-10-7228-8301

Email: seongsy@snu.ac.kr

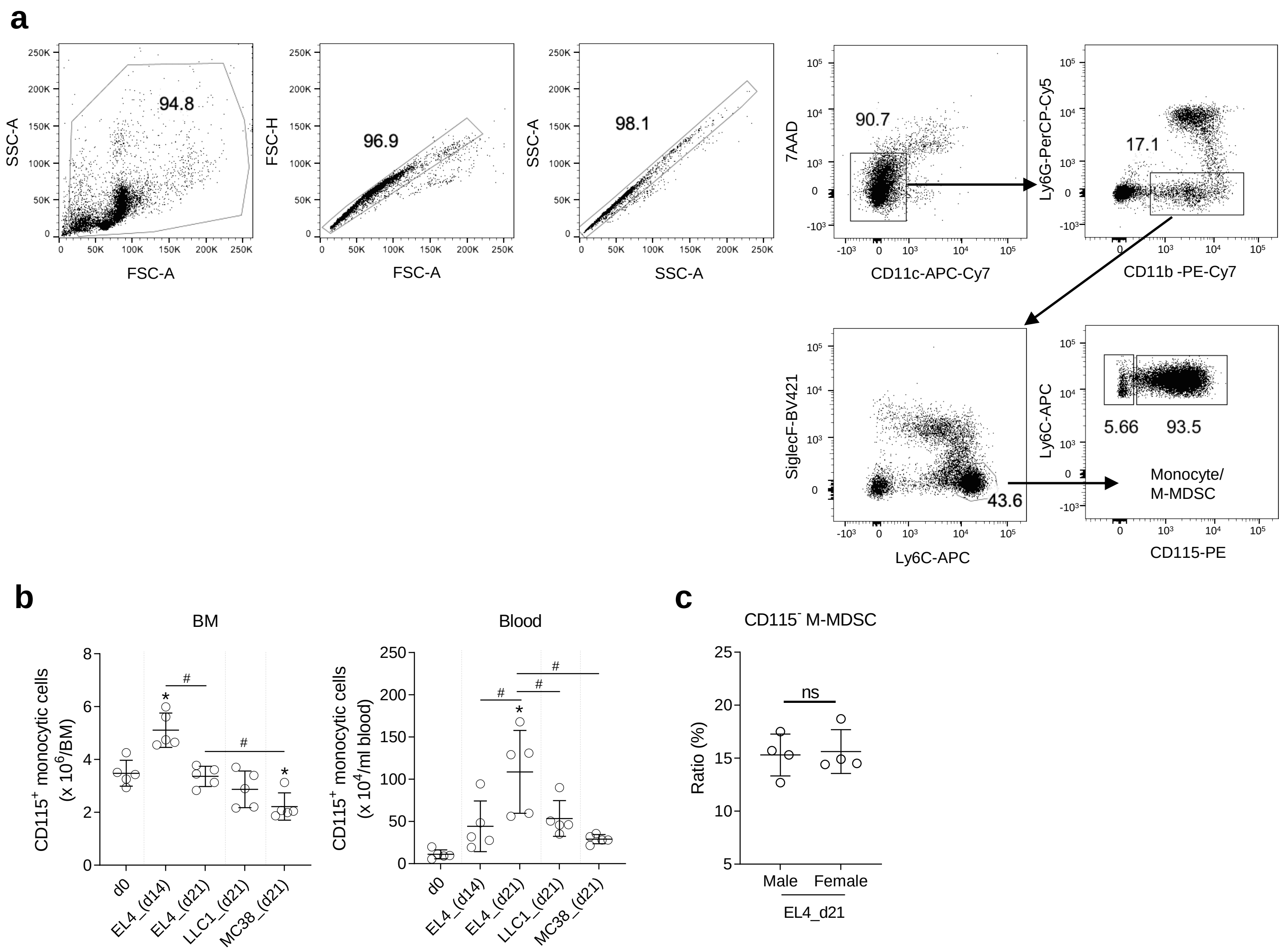

Supplementary Figure 1. $CD115^-$ and $CD115^+$ monocytic cells in naive and TB mice. a Gating strategy used in flow cytometry analysis for monocytic cells ($CD11c^-CD11b^+Ly6G^-SiglecF^-Ly6C^{hi}$). **b** Cell counts for $CD115^+$ in BM and blood of indicated TB mice (n = 5 per group). **c** Ratio of $CD115^-$ M-MDSC in male and female EL4 TB mice after 21 days of tumor inoculation (n = 4 per group). Comparisons between two groups were analyzed using a two-tailed Student's t-test. One-way ANOVA with correction for multiple comparisons test was used for more than two groups comparisons: *$p < 0.05$, all vs. day 0 (d0); $^{\#}p < 0.05$. Data represent mean ± SD.

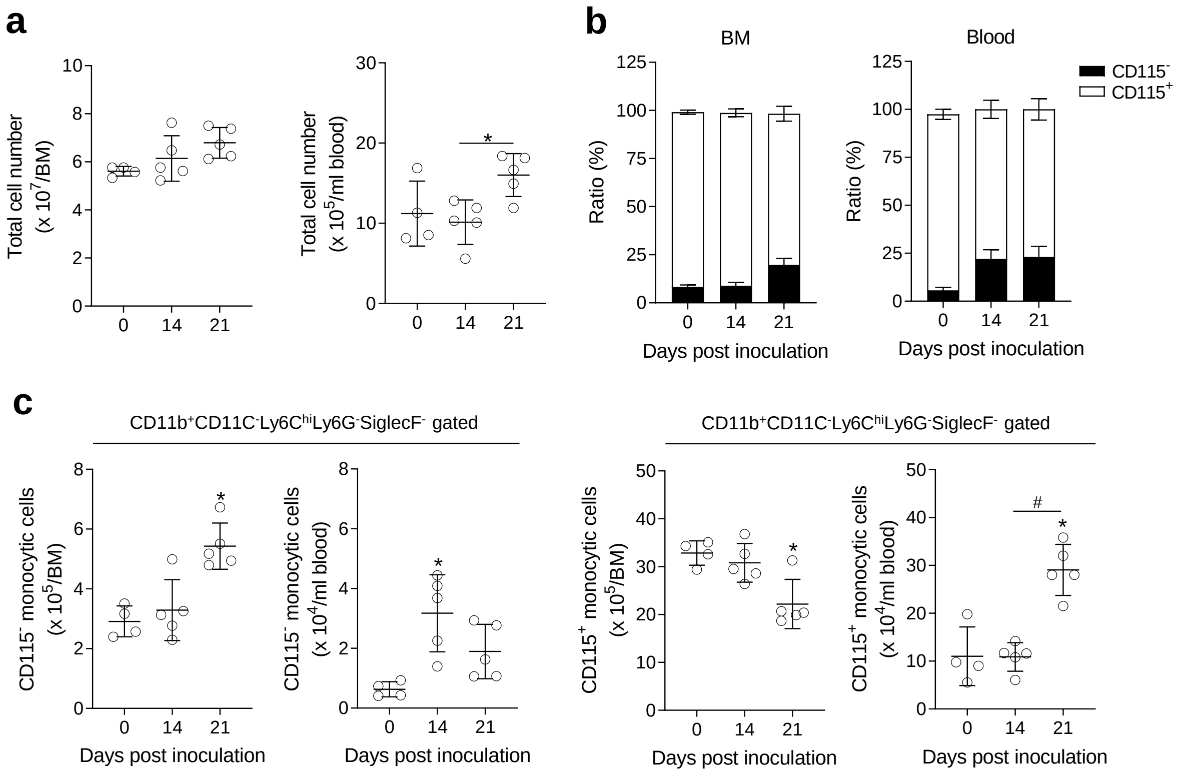

Supplementary Figure 2. Changes in CD115$^-$ and CD115$^+$ monocytic cells during tumorigenesis in MC38 TB mice. a Total BM and blood cell counts at indicated days after MC38 inoculation (n = 4 - 5 per group). **b** Frequency of live CD115$^-$ and CD115$^+$ monocytic cells in BM and blood from MC38 TB mice (n = 4 - 5 per group). **c** Absolute numbers of live CD115$^-$ and CD115$^+$ monocytic cells in BM and blood from MC38 TB mice (n = 4 - 5 per group). One-way ANOVA with correction for multiple comparisons test was used: *$p < 0.05$, all vs. day 0 (d0); $^{\#}p < 0.05$. Data represent mean ± SD.

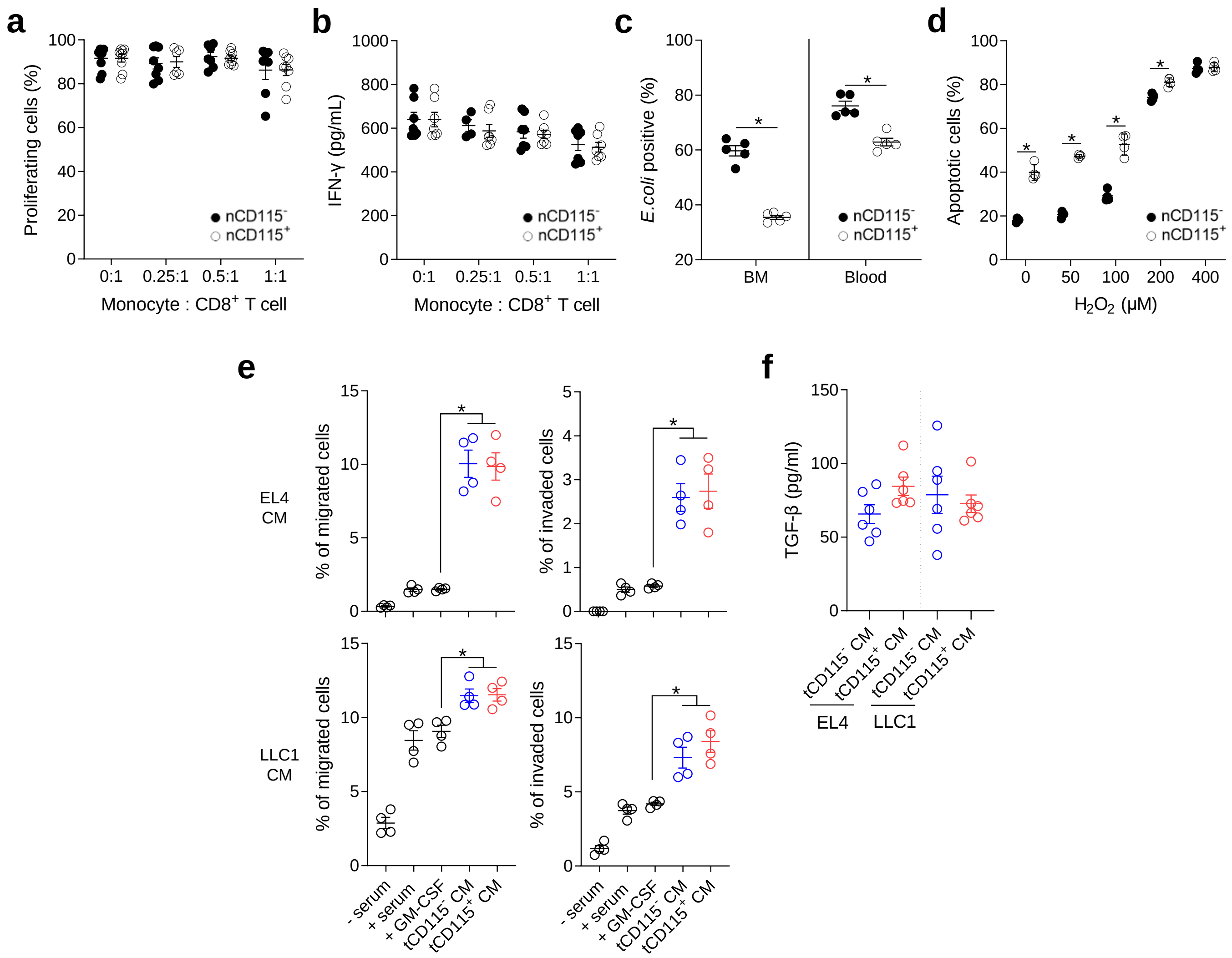

Supplementary Figure 3. Functional phenotypes of CD115$^-$ and CD115$^+$ monocytes. a, b Suppression of CD8$^+$ T cell proliferation by CD115$^-$ and CD115$^+$ naïve monocytes assessed 3 days after co-culture. **a** Ratios of proliferated CD8$^+$ T cells (n = 6 - 9, data are pooled from three independent experiments) and **b** IFN-γ in cell culture supernatant (n = 4 - 7, data are pooled from two to three independent experiments). The letter "n" indicates that cells were isolated from tumor-free mice. **c** BM or blood monocytic cells from naïve and EL4 TB mice (n = 5, each) were incubated with FITC-labelled *E. coli* and the ratios of FITC-positive CD115$^-$ and CD115$^+$ monocytic cells were assessed by FACS. **d** Comparison of apoptosis of BM CD115$^-$ and CD115$^+$ populations in naïve mice (n = 4, each) under various H_2O_2 concentrations. Annexin-V single positive and Annexin-V/7-AAD double-positive cells were sub-gated from CD115$^-$ or CD115$^+$ cells. **e** Percent migration and invasion of EL4 or LLC1 cells with the indicated medium (n = 4, data are pooled from two independent experiments). **f** TGF-β levels in CM (n = 6, data are pooled from two independent experiments). CM was prepared by culturing CD115$^-$ and CD115$^+$ M-MDSC (sorted from EL4 or LLC1 TB mouse) for three days in RPMI complete medium supplemented with GM-CSF (10 ng/ml). Statistical comparisons were performed by Bonferroni's test-corrected ANOVA to compare multiple groups: *$p < 0.05$. Data are mean ± SD.

Supplementary Figure 4 (Zou et al.)

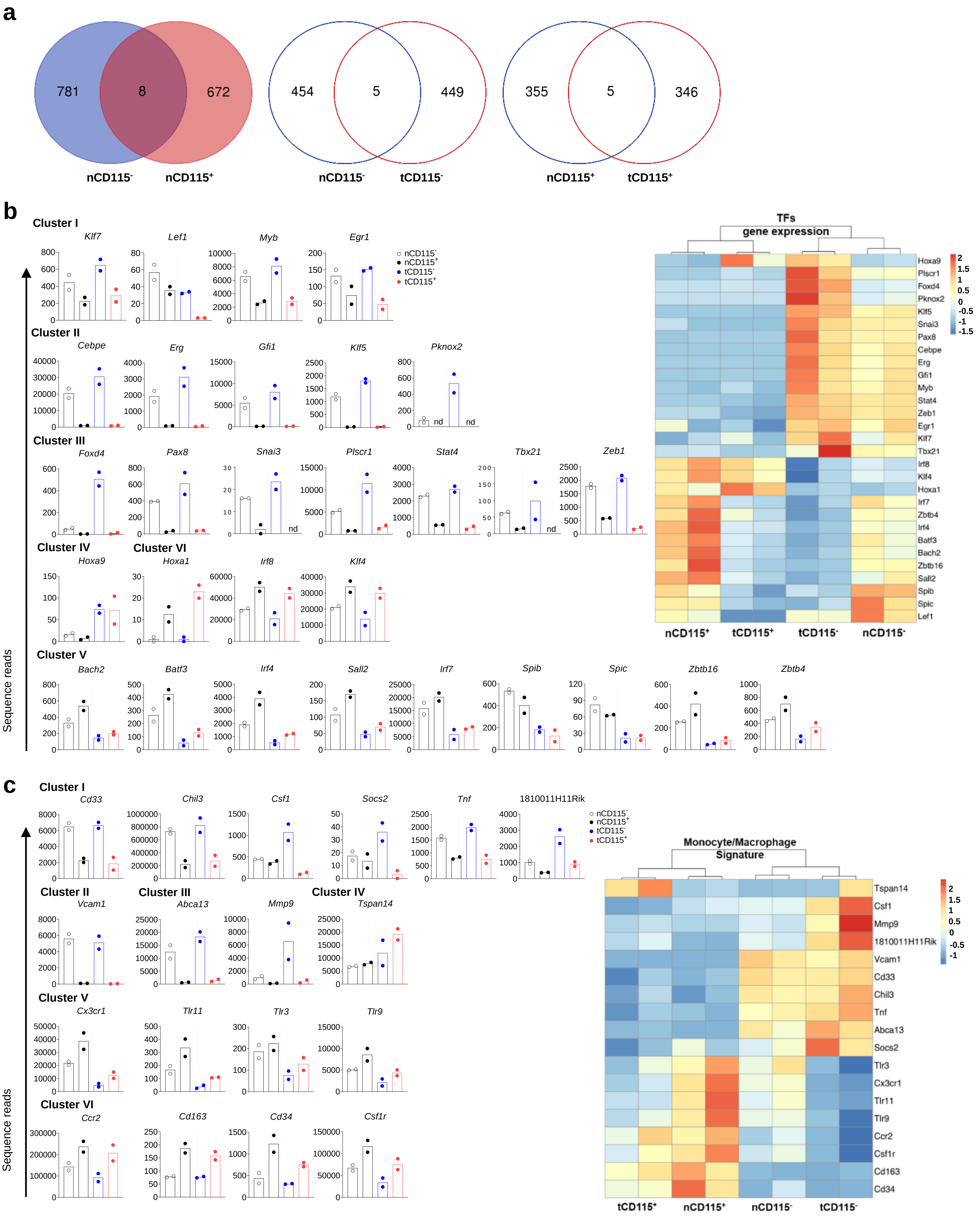

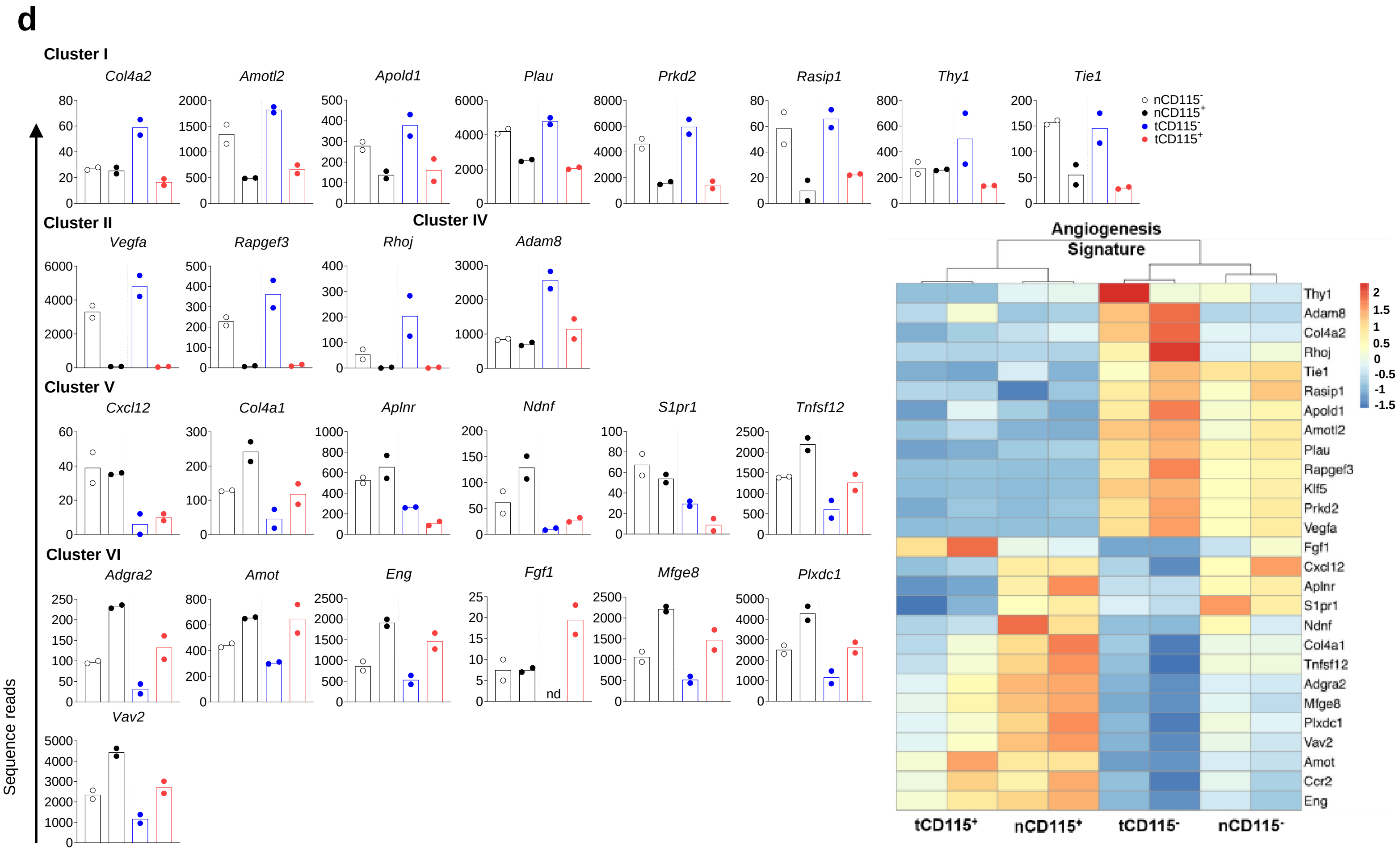

Supplementary Figure 4. Transcriptomic profile of CD115- and CD115+ monocytic cells. a Venn diagram shows variation between sorted CD115- and CD115+ monocyte/M-MDSC libraries from BM of naïve or EL4 TB mice. The letter “n” and “t” in front of CD115 indicate that cells were isolated from tumor-free and TB mice, respectively. **b-d** Distinct transcriptional profiles in gene sets and heatmap related to transcription factors (**b**), monocytes/macrophages (**c**), and angiogenesis (**d**). Data are mean (n = 2 biological replicates).

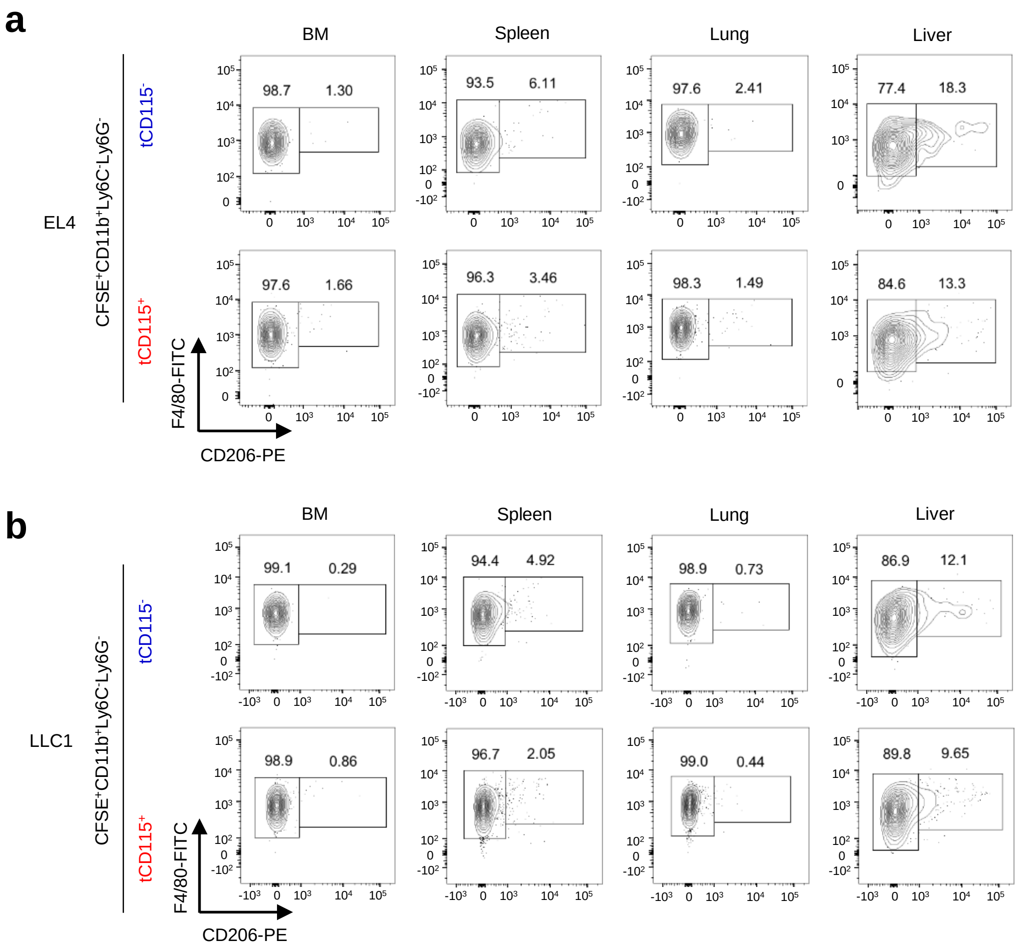

Supplementary Figure 5. Differentiation of CD115^{-} and CD115^{+} M-MDSCs *in vivo*. a, b CD115^{-} and CD115^{+} M-MDSCs were harvested from EL4 or LLC1 TB mice and transferred to recipient TB mice after CFSE labelling as shown in **Fig. 4a**. CFSE^{+} cells were subgated for macrophage analysis. The letter “t” indicates that cells were isolated from TB mice.

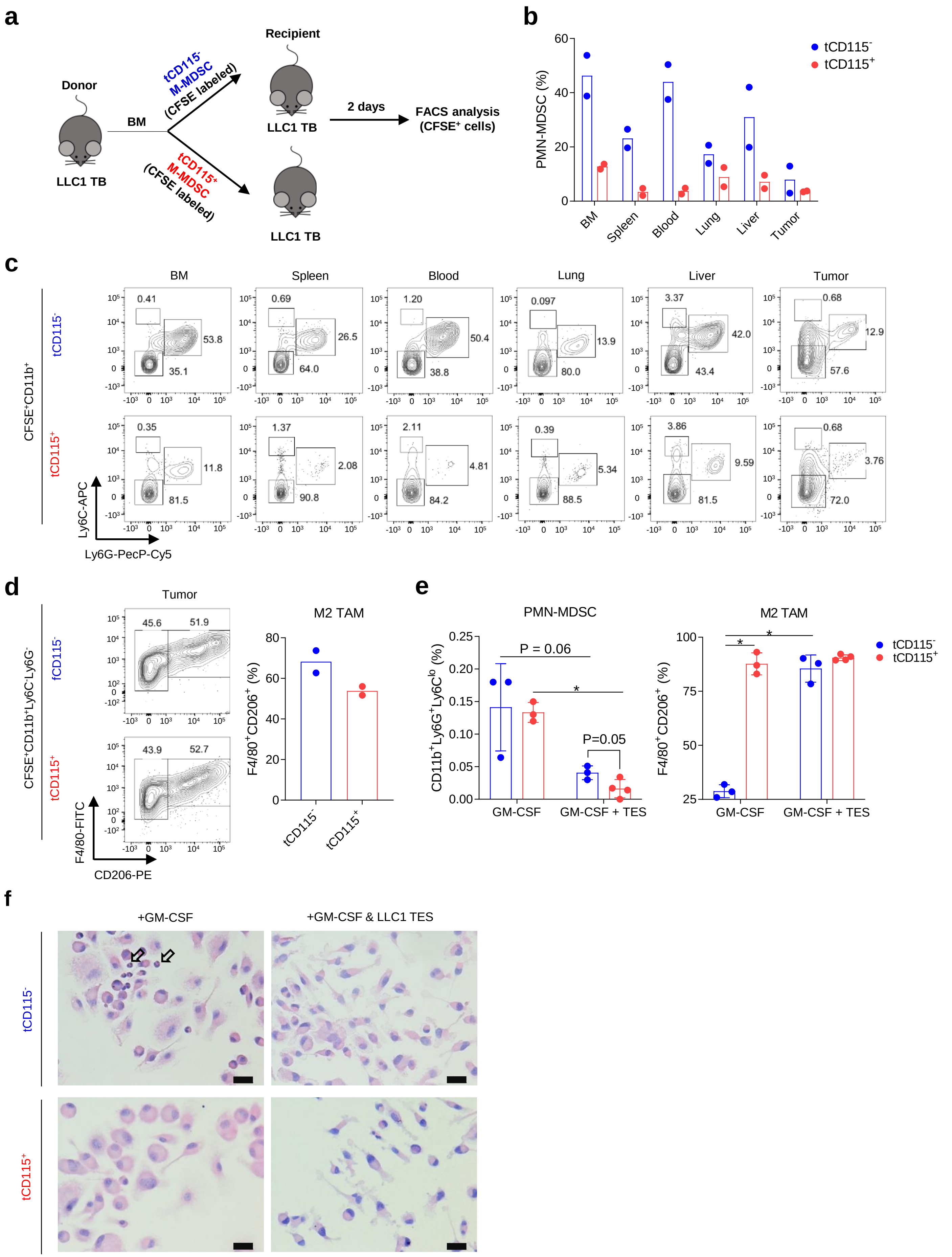

a
Donor
LLC1 TB
BM
tCD115- M-MDSC (CFSE labeled)
tCD115+ M-MDSC (CFSE labeled)
Recipient
LLC1 TB
LLC1 TB
2 days
FACS analysis (CFSE+ cells)
b
PMN-MDSC (%)
tCD115-
tCD115+
BM
Spleen
Blood
Lung
Liver
Tumor
c
BM
Spleen
Blood
Lung
Liver
Tumor
CFSE+CD11b+
tCD115-
tCD115+
Ly6C-APC
Ly6G-PecP-Cy5
d
Tumor
CFSE+CD11b+Ly6C-Ly6G-
F4/80-FITC
CD206-PE
M2 TAM
F4/80+CD206+ (%)
e
PMN-MDSC
P = 0.06
P=0.05
CD11b+Ly6G+Ly6Clo (%)
GM-CSF
GM-CSF + TES
M2 TAM
F4/80+CD206+ (%)
f
+GM-CSF
+GM-CSF & LLC1 TES

Supplementary Figure 6. M-MDSC preferentially differentiate into PMN-MDSC outside the LLC1 tumor mass. a Schematic diagram of experiment design. CFSE-labeled $CD115^-$ or $CD115^+$ M-MDSCs sorted from LLC1 TB mice were intravenously injected into LLC1 TB recipient mice. Transplanted cells ($CFSE^+$) were analyzed by flow cytometry 2 days after injection. The letter “t” indicates that cells were isolated from TB mice. **b-d** Two separate experiments were conducted on EL4 TB mice (n = 3 recipients per group) receiving either $CD115^-$ or $CD115^+$ M-MDSCs, the $CFSE^+$ PMN-MDSC or TAM in indicated tissue from three recipients of each experiment is then analyzed. **b** Ratios of PMN-MDSC ($CD11b^+Ly6G^+Ly6C^{lo}$) differentiation at indicated sites and **c** Representative plots of transplanted $CD115^-$ and $CD115^+$ M-MDSC. **d** Representative plots and ratios of M2 TAM in tumor. **e**, **f** $CD115^-$ and $CD115^+$ M-MDSCs were sorted and differentiated *in vitro* in supplement with GM-CSF (10 ng/ml) ± 5% LLC1 TES for 6 days. Ratios of PMN-MDSCs and M2 TAM (**e**) and a representative H&E stained image (**f**) after *in vitro* culture. Data are pooled from two separated experiments (n = 3 - 4). Arrows indicate PMNs. Scare bar = 20 μM. Statistical comparisons were performed using multiple unpaired Student’s t-test: $*p < 0.05$. Data represent mean or mean ± SD.

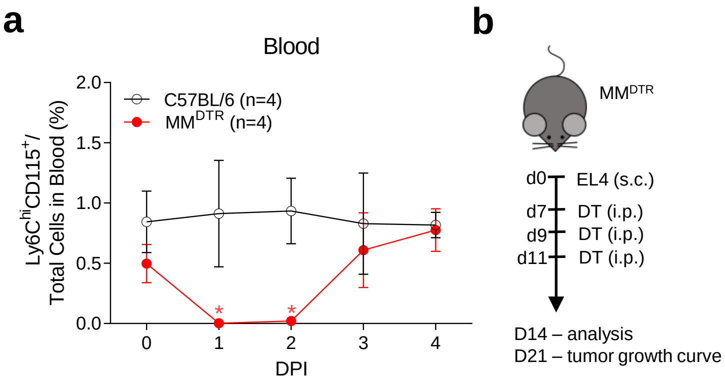

Supplementary Figure 7. Depletion of CD115$^+$ monocytic cells in MM^{DTR} mice. a CD115$^+$ monocytes in blood after intraperitoneal DT injection (10 ng/g body weight). **b** Schematic diagram of CD115$^+$ M-MDSC depletion schedule. 2.5 x 10^5 EL4 tumor cells were subcutaneously injected; DT was injected three times at indicated time points. One-way ANOVA with correction for multiple comparisons test was used: *$p < 0.05$. Data represent mean ± SD.

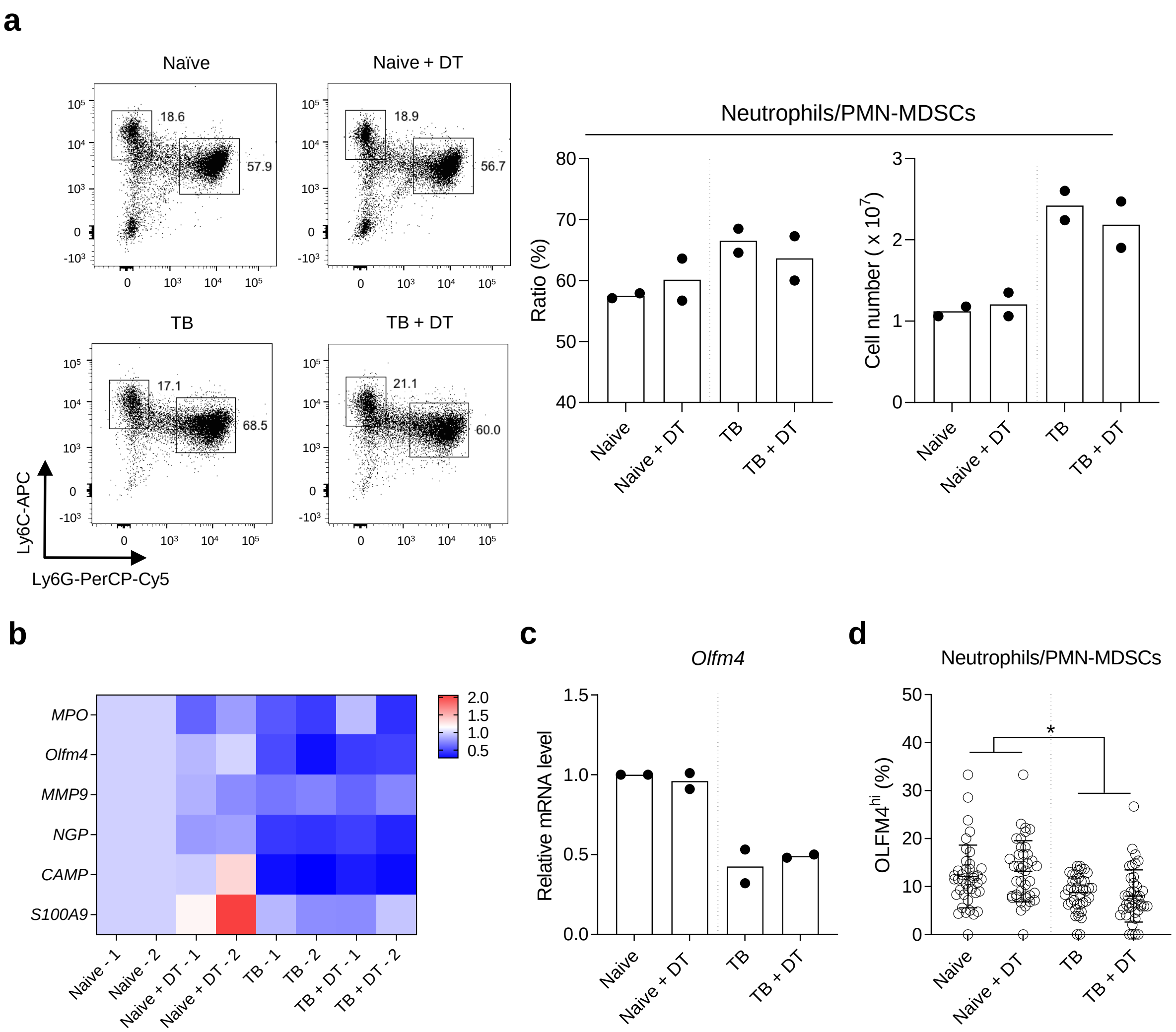

Supplementary Figure 8. The number of neutrophils in C57BL/6 mice is not affected by DT injection. a-d C57BL/6 mice (naïve and EL4 TB) were received intraperitoneal DT injection (10 ng/g body weight) as performed in MM^{DTR} mice. **a** Representative plots of neutrophilic cells in BM of naïve and EL4 TB C57BL/6 mice with or without DT injection. Ratio and absolute numbers of neutrophilic cells in BM of naïve and EL4 TB mice with or without DT injection are shown in graphs. Data shown are from two biological replicates of each group. **b, c** Heatmap of neutrophil-associated gene expression (**b**) and *Olfm4* expression (**c**) in sorted neutrophilic cells from indicated mice. Data shown are from two biological replicates of each group. **d** IHC assessment of ratio of OLFM4^{hi} neutrophilic cells in BM of indicated mice. OLFM4^{hi} cells were counted from twenty random fields. Data pooled from two separate experiments. Statistical comparisons were performed using unpaired Student's *t-test* when only two groups were compared or by Bonferroni's test-corrected ANOVA when more than two groups were compared: *$p < 0.05$. ns; not significant. Data are mean or mean ± SD.

Supplementary Table 1. Primers used in this study.

Genes	Primer sequences	
	**Forward**	**Reverse**
MPO	TCCCACTCAGCAAGGTCTT	TAAGAGCAGGCAAATCCAG
MMP9	TAAGAGCAGGCAAATCCAG	GTGTACACCCACATTTGACG
NGP	AGACCTTTGTATTGGTG	GGTTGTATGCCTCTATGGCTCTA
CRAMP	GCTGTGGCGGTCACTATCAC	TGTCTAGGGACTGCTGGTTGA
S100A9	ATACTCTAGGAAGGAAGGACACC	TCCATGATGTCATTTATGAGGGC
Olfm4	GCCACTTTCCAATTTCAC	GAGCCTCTTCTCATACAC
GAPDH	TGAAGCAGGCATCTGAGGG	CGAAGGTGGAAGAGTGGGAG
