# Supplementary material for: CD115− monocytic myeloid-derived suppressor cells are precursors of OLFM4high polymorphonuclear myeloid-derived suppressor cells
Source: Commun Biol. 2023 Mar 15;6:272. doi: 10.1038/s42003-023-04650-3 (PMC10017706; doi:10.1038/s42003-023-04650-3)
Supplement: Supplementary file 3 — Description of Additional Supplementary Data [file 42003_2023_4650_MOESM3_ESM.docx]

**Description of Additional Supplementary Files**

**File name:** Supplementary Data 1

**Description:** The source data generated during the study.
